# No proportional relationship between shape and size of the femoral canal and the external proximal femur morphology in elderly patients

**DOI:** 10.1016/j.jor.2025.01.018

**Published:** 2025-01-15

**Authors:** Hidde D. Veldman, Ide C. Heyligers, Tim A.E.J. Boymans

**Affiliations:** a Zuyderland Medical Center Heerlen, Dept. of Orthopaedic Surgery and Traumatology, H. Dunantstraat 5, NL, 6419 PC, Heerlen, the Netherlands; b Maastricht University Medical Center, Dept. of Orthopaedic Surgery, P. Debyelaan 25, NL, 6229 HX, Maastricht, the Netherlands; c School of Health Professions Education, Maastricht University, P.O. Box 616, NL, 6200 MD, Maastricht, the Netherlands

**Keywords:** Proximal femur, Morphology, Very elderly, Cementless total hip arthroplasty, Three-dimensional anatomy

## Abstract

**Introduction:**

The proximal femur morphology changes with age, which may complicate the compatibility of contemporary cementless stem designs in very elderly patients. This study investigated the internal and external proximal femur morphology, correlated canal dimensions with external dimensions, and examined whether age-associated changes in the femoral canal and external morphology are related in subjects aged 80 years and older.

**Methods:**

Three-dimensional models of human femora were reconstructed from computed tomographic (CT) scans of 90 very elderly subjects (mean 84 years, range 80–105 years). Morphological parameters describing the location of the femoral head center (FHC) (i.e. neck-shaft angle [NSA], mediolateral offset [ML-offset], and distance between lesser trochanter (LT) and FHC [LT-FHC]) and parameters describing the canal morphology (i.e. the cortices, canal dimensions, and canal flare index [CFI]) were measured. Regression and correlation analyses were performed in order to assess the relation between internal and external morphology.

**Results:**

No significant associations regarding dimensions nor geometry between internal and external femur morphology could be detected. Canal dimensions were not able to predict the external dimensions more accurately than the deviation between the individual value and the mean value for the total cohort.

**Conclusions:**

Based on these findings, proportional sizing of the cementless femoral component is not necessarily endorsed in very elderly patients, and age-associated changes of the femoral canal and external morphology do not appear to be related. However, further research is needed to evaluate the ability of contemporary non-modular cementless stems to anatomically reconstruct the proximal femur in very elderly patients specifically.

## 1 Introduction

A major goal in total hip arthroplasty (THA) is accurate reconstruction of native hip joint biomechanics. Inaccurate reconstruction of the external dimensions of the proximal femur may lead to complications such as leg length discrepancy, joint dislocation, impaired abductor strength, increased wear and patient dissatisfaction.^1^, ^2^, ^3^, ^4^ A cementless femoral component should simultaneously obtain firm fixation in the femoral canal in order to achieve high implant longevity.^5^

Since the size and shape of the proximal femoral canal (the internal femoral morphology) dictate the size and positioning of the femoral component in cementless fixation, the stem should be designed in such way that it enables adequate primary fixation and reconstruction of the external morphology of the proximal femur as well. A wide range of cementless stem designs based on different philosophies are currently available.^5^ Contemporary femoral components are frequently proportionally sized, meaning that the dimensions of the intramedullary part are correlated with those of the extramedullary part (i.e., neck length and the location of the center of rotation). It is unknown whether such proportional relationship is present in the native elderly femur.

The size and morphology of the proximal femur, both intra- and extramedullary dimensions, change with advancing age. Thinning of the cortical bone during ageing leads to a widened femoral canal and less canal flaring in elderly femora, especially in those of (postmenopausal) females.^6^, ^7^, ^8^, ^9^, ^10^ Regarding the external morphology, the femoral head center shifts in a mediocaudal direction during the ageing process.^7^^,^^10^, ^11^, ^12^, ^13^ In very elderly patients, cementless hip arthroplasty results in more implant-related complications when compared to cemented fixation.^14^, ^15^, ^16^, ^17^ This might (partly) be explained by the described anatomical changes and the subsequent mismatch with current cementless stem designs. However, it is also not known whether the age associated changes observed in the internal and external femur morphology are correlated.

This study aims to describe the internal and external proximal femur morphology, and investigates whether there is a proportional relationship between size of the femoral canal and the external femur morphology in very elderly males and females (≥80 years). This could provide insights relevant for femoral component design for this specific age group. Furthermore, this study aims to investigate whether previously described age-associated changes in external femur morphology (i.e. a decreasing neck shaft angle, an increasing mediolateral offset and a decreasing head height) are correlated with separately published age-associated changes in internal femur morphology (i.e. widening of the femoral canal and a decreased canal flaring). If these age-associated phenotypes are correlated in subjects of older age, these might be the symptoms of identical, yet unknown, mechanisms that may take place in individuals during ageing.

## 2 Material and methods

### 2.1 Design

A cross-sectional study design was employed for this anatomical investigation. Three-dimensional (3D) computer models of femora derived from healthy human subjects were studied.

### 2.2 Subjects

A total of 90 Caucasian subjects (50 males, 40 females) aged 80 years or older (mean age 84 years, range 80–105) were studied. From each subject, a computed tomography (CT) scan of the right femur was obtained. These scans were made as an extension of a separate CT scan, made for an unrelated medical reason. Subjects with previous trauma, surgery or bone pathology were excluded from this study. The Sensation Open scanner (Siemens AG, Erlangen, Germany) was used for performing the scans with a field of view of 500 mm and a slice thickness of 1 mm. Each image consisted of 512 x 512 pixels of 0.98 mm × 0.98 mm in size.

### 2.3 Three-dimensional reconstructions

The CT data were loaded into Materialise Mimics (version 10.01, Materialise, Leuven, Belgium). Based on the radiographic density in Hounsfield Units (HU), cortical bone segmentation was performed. Based on Rathnayaka et al.,^18^ each femur was divided into four regions with different minimal HU threshold values for optimal selection of the cortical bone: 1. Head/neck region reached until 1 mm below the femoral head and was segmented with a lower limit of 226 HU, 2. Proximal metaphysis until 30 mm below the lesser trochanter according to the patient-specific 50 % threshold method as described by Hangartner et al.,^19^ 3. Diaphysis reached until 20 mm proximal to the patella with a lower limit of 662 HU, and 4. Distal femur with a lower limit of 226 HU. The four parts were merged into a solid femur model and loaded into a computer aided design (CAD) environment (Rapidform 2006, Inus Technology, Rock Hill, South Carolina) for identification of bony landmarks and measurements of outcome parameters.

### 2.4 Identification of bony landmarks and reconstruction of axes

The femoral head center (FHC) was defined as the geometric center of a sphere fitted on the femoral head by selecting the femoral head. Subsequently, an XYZ-coordinate system with the FHC as origin according to the definitions of the Standardization and Terminology Committee of the International Society of Biomechanics was created.^20^ Anatomically, the axes were defined as follows: X-axis = ventral-dorsal direction, Y-axis = proximal-distal direction, and Z-axis = medial-lateral direction. The reconstruction of the proximal femur axis (PFA) and the femoral neck axis (FNA) was based on circumscribing the cortical bone with circles and connecting the centroids of these circles, as described in detail previously.^11^

**Fig. 1:**
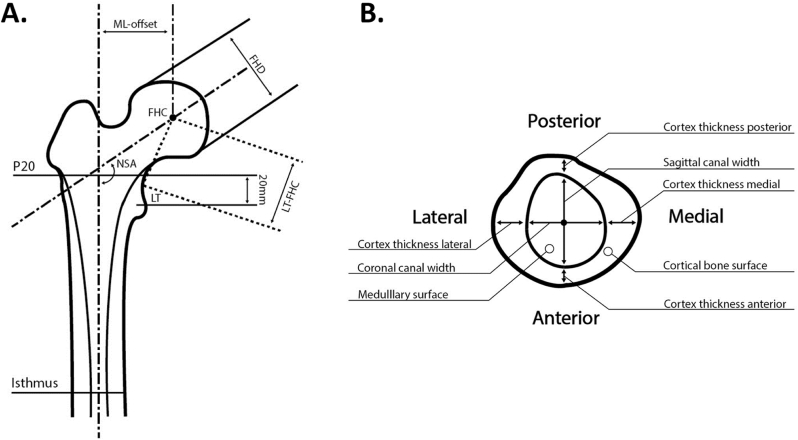
Schematic overview of (A) the outcome parameters describing the external morphology of the proximal femur and the cross-section levels at which the internal parameters were measured and (B) the outcome parameters that were measured at each cross-section level. FHC, femoral head center; LT, lesser trochanter; FHD, femoral head diameter; NSA, neck-shaft angle; ML-offset, mediolateral-offset; LT-FHC, absolute distance between lesser trochanter and the center of the femoral head; P20 represents the crosssection level located 20mm proximal to the lesser trochanter level; Isthmus represents the crosssection level located at isthmus level.

All measurements were performed in Rapidform. Fig. 1 contains a schematic overview of the studied outcome parameters describing the external and internal morphology of the proximal femur.

### 2.5 Parameters describing the external morphology

The neck-shaft angle (NSA) was defined as the angle between the PFA and the FNA in a coronal plane parallel to the FNA. The mediolateral offset (ML-offset) is the mediolateral distance between the PFA and the FHC. As a measure of head height relative to the lesser trochanter (LT), the absolute distance between the LT and the FHC (LT-FHC) was measured with the LT being at the junction between the proximal aspect of the lesser trochanter and the proximal femoral metaphysis. The femoral head diameter (FHD) was defined as the diameter of the sphere that fitted the femoral head.

### 2.6 Parameters describing the internal morphology

At the level 20 mm proximal to the lesser trochanter (P20) and at isthmus level, several measurements on the cross-section of the femur were performed. The cross-sections are perpendicular to the PFA. The lesser trochanter level was defined at the level where the lesser trochanter was most prominent. On both levels, the surface area of the medullary canal and of the cortical bone were measured. Subsequently, the percentage cortical bone surface area of the total periosteal surface area was calculated for that level. The ‘coronal canal width’ is the dimension of the medullary canal through the center of the medullary canal parallel to the Z-axis, while the ‘sagittal canal width’ was the dimension of the medullary canal measured parallel to the X-axis. The cortical thickness was measured at the extensions of those lines in the medial, lateral, anterior and posterior direction.

In order to assess the meta-diaphyseal taper of the proximal femoral canal, we adopted the Canal Flare Index (CFI) as described by Noble et al.^6^ In our study, the ratio between coronal canal width at P20 and at isthmus level was considered CFI_coronal_, while these ratios of sagittal canal width and the medullary surface area between those levels were considered CFI_sagittal_ and CFI_medullary surface_ respectively. Based on the CFI_coronal_, femora could be classified as ‘stovepipe’ (CFI <3.0), ‘normal’ (CFI 3.0 to 4.7), or ‘champagne-flute’ (CFI >4.7) shaped.^6^

### 2.7 Analyses

The mean and standard deviation (SD) of each parameter were calculated. Data was checked for normality using the Shapiro-Wilk test and equality of variances was checked with the Levene's test. The statistical significance of differences between males and females were calculated with the independent samples' *t*-test for normally distributed parameters, or the Mann-Whitney *U* test in case values were not normally distributed. Linear regression analysis was performed between internal and external dimensions. Subsequently, regression equations were used to evaluate how accurate each internal dimension could predict the external dimensions. For examining the correlations between internal and external morphological measures, the Pearson's correlation coefficients (R) were calculated. The threshold for statistical significance was set at p < 0.05. Inter- and intraobserver reliability were calculated based on a random subsample of 30 cases, the Pearson's correlation coefficients were 0.80 and 0.85, respectively. The statistical analyses were performed in SPSS version V23 (IBM Statistics Chicago, IL).

### 2.8 Ethics

The local institutional review board approved this study (approval number 07-T-44/IIIb) and informed consent was obtained from all patients.

## 3 Results

### 3.1 Demographics

Patient characteristics and morphological findings were listed (Table 1).

**Table 1:** Patient characteristics, and parameters describing the external and the internal morphology of the proximal femur.

	Male (n = 50)		Female (n = 40)		P-value
	Mean ± SD	Range	Mean ± SD	Range	
***Patient characteristics***					
Age (years)	83.8 ± 4.1	80.0 to 105.0	84.6 ± 2.9	80.0 to 90.0	0.312
Body height (cm)	172.9 ± 7.0	160.0 to 190.0	161.1 ± 6.9	145.0 to 175.0	**<0.001**
Body weight (kg)	73.6 ± 9.4	50.0 to 95.0	64.8 ± 11.2	40.0 to 80.0	**<0.001**


***External morphology***					
NSA (°)	125.9 ± 5.0	116.3 to 137.5	124.2 ± 6.0	110.9 to 140.3	0.152
ML-offset (mm)	46.4 ± 5.8	33.4 to 59.5	44.2 ± 5.4	26.4 to 53.5	0.070
LT-FHC (mm)	59.5 ± 5.8	45.5 to 75.1	54.9 ± 6.3	42.5 to 72.8	**<0.001**
FHD (mm)	49.9 ± 2.0	45.8 to 54.2	44.1 ± 2.7	38.3 to 51.5	**<0.001**


***Internal morphology***					
*P20 level*					
Medullary surface area (cm^2^)	14.7 ± 2.2	10.7 to 21.2	12.6 ± 1.9	8.8 to 15.9	**<0.001**
Cortical bone surface area (cm^2^)	4.8 ± 1.0	3.4 to 7.1	4.5 ± 1.2	2.4 to 8.7	0.065
Percentage cortical bone (%)	25.0 ± 5.1	15 to 38.6	26.5 ± 6.1	16.8 to 40.0	0.398
Coronal canal width (mm)	48.3 ± 4.8	38.2 to 61.9	44.8 ± 4.8	35.1 to 54.7	**<0.001**
Sagittal canal width (mm)	37.8 ± 3.5	32.1 to 46.4	34.7 ± 3.0	27.1 to 41.0	**<0.001**
Cortex thickness medial (mm)	6.1 ± 1.9	2.5 to 11.8	5.5 ± 1.6	2.2 to 9.6	0.126
Cortex thickness lateral (mm)	2.9 ± 1.0	1.5 to 5.2	3.0 ± 1.2	1.3 to 5.6	0.981
Cortex thickness anterior (mm)	3.3 ± 0.9	1.5 to 5.9	3.7 ± 1.2	1.0 to 7.1	0.091
Cortex thickness posterior (mm)	2.3 ± 1.0	1.2 to 4.4	2.3 ± 1.2	1.1 to 6.6	0.553

*Isthmus level*					
Medullary surface area (cm^2^)	2.0 ± 0.6	1.2 to 3.9	2.3 ± 0.7	1.4 to 4.4	**0.033**
Cortical bone surface area (cm^2^)	5.3 ± 0.6	4.2 to 6.6	3.9 ± 0.7	2.2 to 5.5	**<0.001**
Percentage cortical bone (%)	72.5 ± 6.6	52.6 to 83.2	62.7 ± 9.7	33.9 to 78.1	**<0.001**
Coronal canal width (mm)	14.5 ± 2.0	11.7 to 20.9	14.9 ± 2.1	11.6 to 20.5	0.188
Sagittal canal width (mm)	17.5 ± 2.8	13.1 to 24.5	19.2 ± 3.3	13.7 to 29.1	**0.017**
Cortex thickness medial (mm)	7.8 ± 1.1	4.6 to 10.0	6.7 ± 1.2	4.6 to 9.9	**<0.001**
Cortex thickness lateral (mm)	8.6 ± 1.2	5.6 to 11.0	7.1 ± 1.4	4.3 to 10.4	**<0.001**
Cortex thickness anterior (mm)	6.1 ± 1.4	3.5 to 9.9	4.0 ± 1.1	1.2 to 6.2	**<0.001**
Cortex thickness posterior (mm)	8.1 ± 1.9	4.1 to 12.7	5.7 ± 1.7	1.9 to 10.0	**<0.001**


***Canal flare indices***					
CFI_coronal_	3.4 ± 0.5	2.4 to 4.2	3.0 ± 0.4	2.3 to 4.2	**0.001**
CFI_sagittal_	2.2 ± 0.4	1.5 to 2.9	1.9 ± 0.3	1.0 to 2.5	**<0.001**
CFI_medullary surface_	7.6 ± 1.9	3.6 to 11.1	5.8 ± 1.6	2.4 to 8.9	**<0.001**

P-values that are considered statistically significant (<0.05) are displayed bold.

SD, standard deviation; NSA, neck-shaft angle; ML-offset, mediolateral-offset; LT-FHC, absolute distance between lesser trochanter and the center of the femoral head; FHD, femoral head diameter; P20 level, the cross-section level located 20 mm proximal to the lesser trochanter level; CFI, canal flare index.

### 3.2 External morphology

No differences between males and females regarding the NSA and the ML-offset were observed, while LT-FHC and the FHD were larger in males (Table 1). These data of the external morphology of the proximal femur were published previously.^11^^,^^21^

### 3.3 Internal morphology

At the P20 level, the dimensions of the femoral canal were larger in males in both the mediolateral and the anteroposterior direction (Table 1). Consequently, a significantly larger medullary surface area at this level was observed in males (14.7 cm^2^) compared to females (12.6 cm^2^) (p < 0.001). No differences between males and females were observed at this level regarding thickness of the cortical bone in the anterior, posterior, lateral and medial direction, and subsequently equal values were found for both sexes regarding the cortical bone surface area and the percentage of cortical bone of the total periosteal surface area as well (Table 1).

At isthmus level, the dimensions of the femoral canal were, interestingly, equal in males and females in the mediolateral direction, and even 1.7 mm larger in females in the anteroposterior direction (p < 0.001) (Table 1). Accordingly, the medullary canal surface area at this level was observed to be larger in females (2.3 cm^2^) than in males (2.0 cm^2^) (p < 0.001). Cortical bone was thinner in the anterior, posterior, lateral and medial direction in females, resulting in lower values for cortical bone surface area and percentage of cortical bone of the total periosteal surface area (Table 1).

The CFI in the coronal and sagittal plane as well as based on the medullary surface area, appeared to be significantly lower in females compared to males (Table 1). This indicates less flaring of the femoral canal in females. Based on the CFI_coronal_, 52.5 % of the female femora was considered to have a ‘stovepipe-shape’, while in males this was only 22.0 %. The remaining femora were considered ‘normal’. No ‘champagne flute-shaped’ femora were present in this cohort.

### 3.4 Relationship between internal and external dimensions

**Fig. 2:**
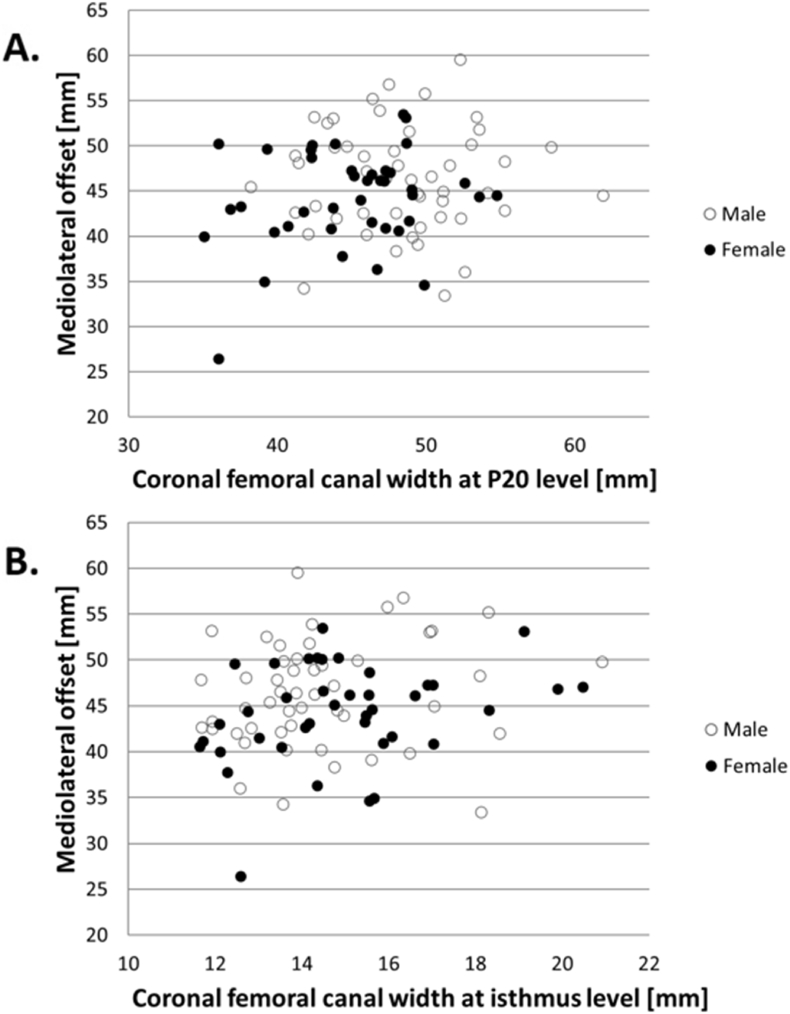
Scatter plots of the mediolateral offset and the coronal canal width (A) at the level 20 mm proximal to the lesser trochanter (P20) and (B) at isthmus level.

The coronal or sagittal canal width at the P20 level and at isthmus level were not related to external parameters describing the location of the FHC (i.e. ML-offset and LT-FHC) because linear regression analyses revealed no significant association (Fig. 2, Table 2). Consequently, the derived equations were not able to predict the external dimensions more accurately than the deviation between the true value and the mean value for the total cohort (Table 2).

**Table 2:** The relation between dimensions of the internal and external proximal femur morphology by the means of linear regression, and the accuracy of predictions by the use of derived equations compared to the average discrepancy with the mean cohort value.

		Linear regression analysis		Accuracy of prediction		
		Significance (p-value)	Equation	Mean difference ± SD [mm]	<6 mm difference [%]	<10 mm difference [%]
ML Offset prediction by linear regression Based on:						
	P20 coronal canal width	NS (0.134)	Y = 0.178 X + 37.072	4.5 ± 3.4	74.7	91.2
	P20 sagittal canal width	NS (0.841)	Y = 0.034 X + 44.169	4.5 ± 3.5	73.6	90.1
	Isthmus coronal canal width	NS (0.084)	Y = 0.505 X + 37.989	4.3 ± 3.6	73.6	91.2
	Isthmus sagittal canal width	NS (0.547)	Y = −0.119 X + 47.562	4.4 ± 3.5	74.7	90.1
ML Offset value versus average ML Offset of total cohort		–	–	4.4 ± 3.5	74.4	91.1


LT-FHC prediction by linear regression Based on:						

	P20 coronal canal width	NS (0.305)	Y = −0.138 X + 63.898	4.9 ± 4.0	68.1	87.9
	P20 sagittal canal width	NS (0.303)	Y = 0.194 X + 50.401	4.8 ± 4.2	65.9	87.9
	Isthmus coronal canal width	NS (0.790)	Y = −0.088 X + 58.754	4.9 ± 4.2	67.0	87.9
	Isthmus sagittal canal width	NS (0.252)	Y = −0.254 X + 62.092	4.9 ± 4.0	68.1	87.9
LT-FHC value versus average LT-FHC of total cohort		–	–	4.9 ± 4.2	67.7	89.9

NS, not significant (i.e. p ≥ 0.05); SD, standard deviation; ML-offset, mediolateral-offset; LT-FHC, absolute distance between lesser trochanter and the center of the femoral head; P20, the.

cross-section level located 20 mm proximal to the lesser trochanter level; Isthmus, the cross-section level located at the isthmus.

Accordingly, no Pearson correlation coefficients describing the relationship between size of the proximal femoral canal and the external geometry of the proximal femur were found to be statistically significant in males nor females (Table 3).

**Table 3:** Pearson correlation coefficients displaying the correlation between parameters describing the internal morphology and the external morphology of the proximal femur. None of these Pearson correlation coefficients is considered statistically significant.

	NSA	ML-offset	LT-FHC
*Internal morphology*			
*P20 level*			
Medullary surface area	M: 0.075 (p = 0.605)	M: 0.032 (p = 0.823)	M: 0.168 (p = 0.242)
	F: 0.167 (p = 0.302)	F: 0.253 (p = 0.115)	F: 0.101 (p = 0.536)
Cortical bone surface area	M: 0.169 (p = 0.240)	M: 0.212 (p = 0.139)	M: 0.140 (p = 0.333)
	F: 0.199 (p = 0218)	F: 0.095 (p = 0.559)	F: 0.259 (p = 0.107)
Percentage cortical bone	M: 0.088 (p = 0.543)	M: 0.149 (p = 0.301)	M: 0.006 (p = 0.969)
	F: 0.043 (p = 0.793)	F: 0.049 (p = 0.764)	F: 0.116 (p = 0.477)
Coronal canal width	M: 0.026 (p = 0.858)	M: 0.009 (p = 0.951)	M: 0.231 (p = 0.106)
	F: 0.156 (p = 0.336)	F: 0.225 (p = 0.162)	F: 0.311 (p = 0.050)
Sagittal canal width	M: 0.009 (p = 0.948)	M: 0.159 (p = 0.271)	M: 0.077 (p = 0.593)
	F: 0.049 (p = 0.763)	F: 0.071 (p = 0665)	F: 0.029 (p = 0.861)

*Isthmus level*			
Medullary surface area	M: 0.055 (p = 0.705)	M: 0.114 (p = 0.430)	M: 0.010 (p = 0.948)
	F: 0.060 (p = 0.712)	F: 0.059 (p = 0.717)	F: 0.009 (p = 0.956)
Cortical bone surface area	M: 0.087 (p = 0.548)	M: 0.161 (p = 0.265)	M: 0.137 (p = 0.342)
	F: 0.003 (p = 0.983)	F: 0.004 (p = 0.980)	F: 0.238 (p = 0.139)
Percentage cortical bone	M: 0.005 (p = 0.972)	M: 0.032 (p = 0.826)	M: 0.036 (p = 0.802)
	F: 0.050 (p = 0.758)	F: 0.046 (p = 0.780)	F: 0.081 (p = 0.619)
Coronal canal width	M: 0.066 (p = 0.647)	M: 0.143 (p = 0.321)	M: 0.030 (p = 0.835)
	F: 0.160 (p = 0.324)	F: 0.294 (p = 0.065)	F: 0.059 (p = 0.718)
Sagittal canal width	M: 0.074 (p = 0.610)	M: 0.088 (p = 0.544)	M: 0.020 (p = 0.892)
	F: 0.208 (p = 0.199)	F: 0.131 (p = 0.419)	F: 0.036 (p = 0.826)

*Canal flare indices*			
CFI_coronal_	M: 0.095 (p = 0.512)	M: 0.143 (p = 0.321)	M: 0.145 (p = 0.315)
	F: 0.007 (p = 0.968)	F: 0.107 (p = 0.512)	F: 0.311 (p = 0.051)
CFI_sagittal_	M: 0.055 (p = 0.703)	M: 0.168 (p = 0.245)	M: 0.056 (p = 0.699)
	F: 0.232 (p = 0.150)	F: 0.197 (p = 0.224)	F: 0.076 (p = 0.641)
CFI_medullary surface_	M: 0.024 (p = 0.867)	M: 0.106 (p = 0.464)	M: 0.127 (p = 0.381)
	F: 0.187 (p = 0.249)	F: 0.115 (p = 0.478)	F: 0.015 (p = 0.927)

P-values that are considered statistically significant (<0.05) are displayed bold.

M, male; F, female; NSA, neck-shaft angle; ML-offset, mediolateral-offset; LT-FHC, absolute distance between lesser trochanter and the center of the femoral head; P20 level, the cross-section level located 20 mm proximal to the lesser trochanter level; CFI, canal flare index.

Furthermore, age-associated changes in internal and external morphology do not seem correlated since a lower NSA, a larger ML-offset and a lower LT-FHC are not accompanied with larger dimensions of the femoral canal or less cortical bone at the P20 level and at isthmus level, nor are they correlated with less canal flaring based on the non-significant Pearson correlation coefficients between these parameters in males and females (Table 3).

## 4 Discussion

This three-dimensional CT-based study on very elderly subjects (≥80 years) describes the internal and external proximal femur morphology, and found no significant proportional relationship between morphology of the femoral canal and the external proximal femur. The external morphological parameters presented in this study have been published previously and indicated a relative varus positioning of the FHC when compared to younger controls,^11^^,^^21^ which is in accordance with other studies.^7^^,^^10^^,^^12^^,^^13^ The studied internal morphological parameters indicate that elderly females had smaller canal dimensions and comparable cortices at P20 level, while having comparable canal dimensions and smaller cortices at isthmus level when compared to elderly males. This is in accordance with previous literature.^6^^,^^7^^,^^9^ Generally, lower values for CFI_coronal_ are seen in the elderly.^6^^,^^7^^,^^9^^,^^10^^,^^22^ Previous studies in subjects with a comparable age reported comparable CFI values.^9^^,^^22^ Clinically, orthopaedic surgeons use larger sized cementless femoral components in older patients in order to enable adequate fixation in the widened canal.^23^

No association between dimensions of the femoral canal and the external dimensions describing the FHC location were found. The individual ML-offset and LT-FHC deviated 4.4 mm and 4.9 mm respectively from the mean cohort values on average, and was within 10 mm in 91.1 % and 89.9 % respectively (Table 2). Based on these findings, a stem design with average dimensions, which are independent from the internal dimensions, could potentially be an equally successful design for this population (Table 2). In clinical practice, minimal discrepancies could be further minimalised by optimizing the positioning of the femoral implant, with lateralized designs, and with neck length/ head correction. The latter are optional in most contemporary implant systems. Meticulous preoperative planning by templating on 2D radiographs^24^ or on 3D CT-scans,^25^ as well as precise intraoperative monitoring, is advocated for optimal reconstruction.

Previous studies described age-associated changes in femoral canal morphology such as canal widening, thinner cortices and less flaring of the canal^6^, ^7^, ^8^, ^9^, ^10^ and an age-associated head shift towards a relative varus position^7^^,^^10^, ^11^, ^12^, ^13^^,^^21^ separately and on group level. It is known that during the ageing process, especially in postmenopausal women, the bone mineral density (BMD) decreases. The relationship between osteoporosis and changes in femur morphology has been described by significant correlations between a decreasing BMD, and a decreasing CFI^26^ or progressive canal widening.^27^ Potentially, the decrease in NSA and the accompanied increase in ML-offset might also be explained by poorer bone mineralization. This was also suggested by Carmona et al., who studied the morphology of the proximal femur on CT-scans of 628 healthy patients.^10^ They found that increasing age was associated with a decrease in CFI and NSA, and an increase in ML-offset.^10^ Bigart et al. studied whether preoperative femoral morphology could predict the risk of postoperative periprosthetic femur fractures after cementless THA.^28^ They compared cases of periprosthetic fractures with matched controls and found in patients with equal age that the NSA and CFI were lower in the group with a periprosthetic femur fracture.^28^ These findings suggest that the morphologically typical elderly femur is a frail femur prone to periprosthetic fractures after cementless THA, independent of the patient's age. By correlating the internal and external morphological parameters in a population of very elderly subjects, we aimed to investigate whether these were the consequence of a common but yet unknown physiological mechanism causing both phenotypical age-associated changes to occur. Based on our analyses, this seemed not to be the case.

Previous studies on the relation between internal and external proximal femur morphology are scarce. Massin et al. studied 200 AP radiographs of subjects with a mean age of 67 years and found significant (p < 0.001), but weak correlations between coronal canal width on different levels and the ML-offset (R = 0.25 to 0.33) or canal width at isthmus level and head height (R = 0.30).^29^ Discrepancies in the results might be attributed to differences in the population's age or the measurement methodology, with 2D radiographs being notably inferior compared to 3D-CT measurements. A more recently performed study by Wegrzyn et al. assessed 3D-CT-scans of 151 human male femora with a mean age of 66 years and found significant correlations between a 3D internal parameter ‘the proximal femoral metaphyseal volume (PFMV)’ and external parameters: ML-offset (R = 0.223, p = 0.001), head height (R = 0.638, p < 0.0001), neck length (R = 0.528, p < 0.0001) and NSA (R = 0.481, p < 0.0001).^30^ The conflicting findings with our results might be explained by the PFMV measure, which was defined as the volume of the femoral canal between 50 and 90 mm distal to the FHC. Since the femoral canal flares, especially in the relatively younger age that was studied, a lower level at the femur will most likely correspond to a lower endomedullary volume. Because the height of the FHC directly dictates the level at which the volume was measured, a relationship between the PFMV and parameters describing the head height (such as NSA, head height, and neck length) is a logical consequence inherent to their methodology. Furthermore, we believe that solely investigating volume is not an optimal measure for internal morphology because dimensions in X and Z direction remain unknown.

Some limitations have to be considered. First, in order to draw more rigid conclusions on the relation between age-associated changes in internal and external proximal femur morphology, a future longitudinal study that correlates the dynamic age-related changes in external and internal morphology by using the change over a time-interval should be performed. Second, this study provides insight in the morphology of the proximal femur in very elderly subjects, but the ability of current stems to reconstruct the native anatomy in this age group remains a topic for future research. Third, in our analyses we chose to thoroughly investigate a set of predefined morphological parameters (Fig. 1). However, not all relevant parameters describing proximal femur morphology nor all canal levels relevant for implant fixation have been studied. Finally, our dataset was limited to 90 Caucasian subjects (50 males and 40 females). Therefore, it remains unknown whether the conclusions would be different for other ethnic populations. Regarding the number of subjects we studied, our total cohort provides the power to detect a Pearson correlation coefficient of 0.3 with 80 % power. Our data reveal Pearson correlation coefficients indicating weak relationships in both males (positive R range: 0.005 to 0.212 and negative R range: 0.006 to −0.275) and females (positive R range: 0.007 to 0.294 and negative R range: 0.003 to −0.311). These weak relationships confirm our conclusion that there is no significant and clinically relevant correlation.

## 5 Conclusions

This study describes the internal and external morphology of the proximal femur in very elderly subjects based on 3D-CT measurements. These values are relevant for femoral component design for the oldest age group. No association between dimensions of the femoral canal and the dimensions describing the FHC location were found in our population. Whether this finding complicates the ability of contemporary non-modular cementless stem designs for adequate fixation and FHC reconstruction simultaneously in very elderly patients should be a topic for future research. However, based on our findings, a non-modular cementless stem design with fixed external dimensions and varying internal dimensions per stem size could achieve comparable accuracy in reconstructing the FHC to that of a design in which both internal and external parameters increase proportionally with stem size. Furthermore, it seems that there is no relation between age-associated anatomical changes in femoral canal morphology such as canal widening, thinner cortices and less flaring of the canal; and the age-associated head shift towards a relative varus position. However, a future longitudinal study investigating age-associated changes of the proximal femur is needed to study this phenomenon in an optimal fashion.

## CRediT authorship contribution statement

**Hidde D. Veldman:** Conceptualization, Methodology, Formal analysis, Investigation, Writing – original draft, All authors have agreed to be held accountable for the content of the work. **Ide C. Heyligers:** Conceptualization, Methodology, Formal analysis, Investigation, Writing – review & editing, All authors have agreed to be held accountable for the content of the work. **Tim A.E.J. Boymans:** Conceptualization, Methodology, Formal analysis, Investigation, Writing – review & editing, All authors have agreed to be held accountable for the content of the work.

## Guardian patient's consent

Written informed consent was obtained from all patients included in this study.

## Ethical statement

The local institutional review board approved this study (approval number 07-T-44/IIIb) and informed consent was obtained from all patients.

## Funding

There was no funding for this project.

## Conflict of interest

The authors declare that the research was conducted in the absence of any commercial or financial relationships that could be construed as a potential conflict of interest.
